# Structural Core-Shell beyond Chemical Homogeneity in Non-Stoichiometric Cu_5_FeS_4_ Nano-Icosahedrons: An in Situ Heating TEM Study

**DOI:** 10.3390/nano10010004

**Published:** 2019-12-18

**Authors:** Bin Zhang, Xiaowei Zhao, Tianrui Dong, Aijuan Zhang, Xiao Zhang, Guang Han, Xiaoyuan Zhou

**Affiliations:** 1 Analytical and Testing Center, Chongqing University, Chongqing 401331, China; seashellemma@163.com (X.Z.); xiaoyuan2013@cqu.edu.cn (X.Z.); 2 College of Materials Science and Engineering, Chongqing University, Chongqing 400044, China; 20182871@cqu.edu.cn (X.Z.); 20182885@cqu.edu.cn (T.D.); guang.han@cqu.edu.cn (G.H.); 3 College of Physics, Chongqing University, Chongqing 401331, China; aijuanzhang@uestc.edu.cn

**Keywords:** core-shell structure, Cu_5_FeS_4_ icosahedral nanoparticles, in situ TEM, thermal stability, intrinsic strain of icosahedron

## Abstract

Thermal stability of core-shell structured nanoparticles is of vital importance to their practical applications at elevated temperature. Understanding the evolution of chemical distribution and the crystal structure of core-shell nanostructures with temperature variation at the nanoscale will open the route for practical applications and property enhancement of nanoparticles through proper design of new nanomaterials. In this study, core-shell non-stoichiometric Cu_5_FeS_4_ icosahedral nanoparticles were investigated by in situ heating transmission electron microscopy. Compared to the high structural and compositional stability at room temperature, the interdiffusion of Cu and Fe atoms became significant, ending up with disappearance of chemical difference in the core and shell over 300 °C. In contrast, different crystal structures of the core and shell were preserved even after heating at 350 °C, indicating the high structural stability. The inconsistency between chemical composition and crystal structure should be ascribed to the interaction between the intrinsic strain existing in the icosahedrons and various structures of this material system. In other words, the geometrically intrinsic strain of the nano-icosahedrons is helpful to modulate/maintain the core-shell structure. These findings open new opportunities for revealing the thermal stability of core-shell nanostructures for various applications and are helpful for the controllable design of new core-shell nanostructures.

## 1. Introduction

In recent years, core-shell nanostructures have attracted extensive attention for catalysis [1], energy conversion/storage [2,3], sensors [4], structure/property modifications [5], and so on because of their outstanding optical and electrical properties. One of the key issues for the materials’ practical applications is stability, especially under high-temperature conditions. With typical sizes down to nanometer scale, the proportion of surface atoms is significantly increased; therefore, nanomaterials often reveal tremendous surface effects but reduced stability compared with bulk materials. Elemental diffusion [6,7], decomposition [8,9], and gas corrosion (such as oxidation [8,10]) more easily occur at elevated temperatures for nanomaterials. Thus, high temperature and related treatment often disturbs/changes the core-shell structures that will, in turn, affect the performance and applications of core-shell nanostructures. Revealing the morphological, structural, and compositional evolution of nanomaterials (e.g., core-shell nanostructures) during the thermal process is of vital importance for both scientific research and practical applications. In situ transmission electron microscopy (TEM) is a powerful platform to observe such evolutions at the nanometer/atomic scale, which provides one of the best solutions for thermal stability studies. For example, the thermal stability and structural reconfiguration of Ni-Co core-shell nanoparticles were investigated through in situ TEM and XPS, which systematically reveals core-shell reconfiguration and is crucial for their utilization under high-temperature conditions [11]. By means of in situ heating TEM, the temperature-dependent diffusion process was studied, and an inversion of the core-shell structure from Ni-Au to Au-Ni was observed at 400 °C [12]. Such findings are valuable for the catalysis application of bimetallic nanoparticles.

Thermoelectric materials [13], usually utilized for electricity generation from waste heat, have potential to tackle the energy crisis and environmental problems [14]. Nanostructures are widely introduced to improve the performance of thermoelectric materials [15,16,17,18], and advanced electron microscopy is frequently conducted to reveal the structure–property relationships [19]. Cu_5_FeS_4_, known as a natural, low-cost mineral (bornite) abundant in the Earth, was reported to be a potential thermoelectric material because of its low thermal conductivity and considerable thermoelectric properties in 2014 [20]. After that, plenty of methods and works [21,22,23] were developed to enhance the thermoelectric properties of this system, and different nanostructures of Cu_5_FeS_4_ were also synthesized successfully [24,25,26,27]. In our previous work, core-shell non-stoichiometric Cu_5_FeS_4_ icosahedral nanoparticles were synthesized by a colloidal solution method and achieved an enhanced thermoelectric figure of merit (zT) of 0.62 at 710 K via twin engineering, which is ~51% larger than the zT of Cu_5_FeS_4_ without multiple twinned structures [27]. One particular finding is that the twinned structures were well maintained after spark plasma sintering (involving a heating process) while the distribution of different elements became homogeneous, indicating different thermal stability between composition and structure of the core-shell particles [27]. As structural (e.g., twinned and crystalline structure) and compositional evolutions under thermal treatment are of vital importance for understanding and improving the performance of such nanoparticles, it is imperative to investigate their thermal stability systematically.

In the present work, ex situ and in situ heating TEM studies were conducted on non-stoichiometric Cu_5_FeS_4_ nano-icosahedrons, in which the structural and chemical evolutions were observed at the nanometer scale. At room temperature (RT), the chemical composition and crystal structure of the nanoparticles were stable (almost unchanged over 30 months exposure in air). At high temperature (over 300 °C), the chemically varied core-shell gradually disappeared (i.e., composition became uniform), while the different crystal structures in core and shell can still be preserved. These observations indicate that the structures of the annealed Cu_5_FeS_4_ nano-icosahedrons did not completely change accordingly with the chemical changes. This phenomenon is thought to mainly be due to the geometrically intrinsic strain present in the icosahedrons. The findings in this work provide direct evidence for the thermal stability of Cu_5_FeS_4_ core-shell nanoparticles and shed light on utilizing intrinsic strain to design/modulate the structure and stability of core-shell nanoparticles.

## 2. Materials and Methods

The procedures for synthesis of Cu_5_FeS_4_ core-shell icosahedral nanoparticles were described in detail in our previous work [27]. Morphology of nanoparticles was characterized by a field-emission environmental scanning electron microscope (SEM, Thermoscientific Quattro S, Brno, Czech) at 5 kV. TEM-related studies, including high-resolution TEM (HRTEM), selected area electron diffraction (SAED), high-angle annular dark field (HAADF), and energy-dispersive X-ray spectroscopy (EDS) mapping, were conducted on a Thermoscientific Talos F200S G2 microscope (Brno, Czech) at 200 kV. Two kinds of TEM samples were prepared. First, the icosahedral nanoparticles were dispersed in ethanol and directly deposited on copper grids. Second, ultramicrotomy was applied to section nanoparticles into thin slices (ca. 30 nm) for HRTEM observation and composition determination for cores and shells, since the nanoparticles with a size of 100–200 nm were too large/thick for these studies. The nanoparticles were firstly embedded in resin (Epon 812, West Chester, PA, USA) after 80 °C heating/solidification for 48 h in vacuum and then sectioned to thin slices via ultramicrotome (Leica EM UC7, Vienna, Austria). The icosahedral nanoparticles and thin slices were transformed to TEM grids for ex situ studies and to heating MEMS chips for further in situ heating TEM studies by using a FEI NanoEx-w/v MEMS holder.

## 3. Results and Discussion

### 3.1. Stability Study at Ambient Temperature

**Figure 1:**
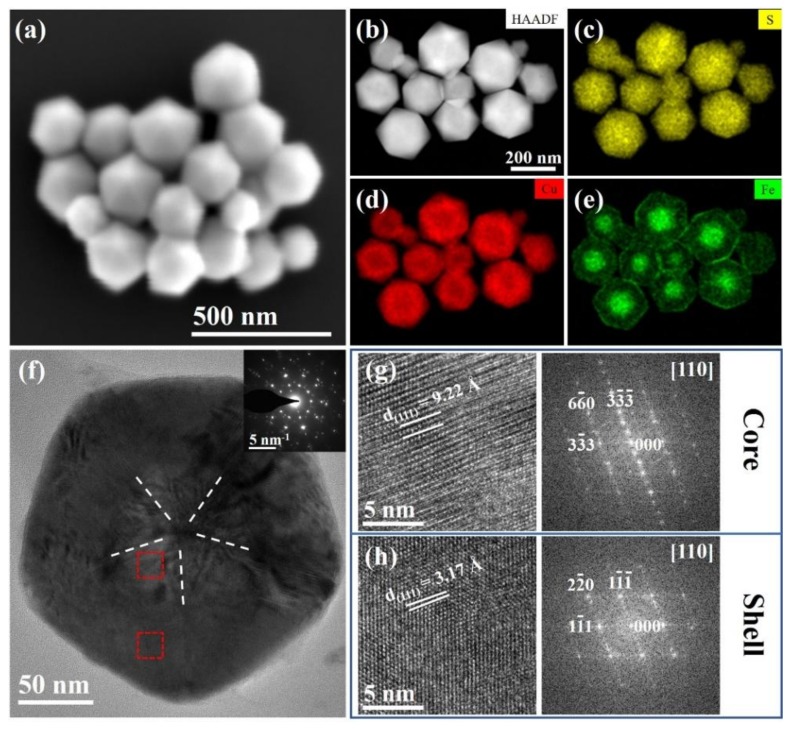
SEM and TEM characterizations of long-term stored Cu_5_FeS_4_ nanoparticles. (**a**) SEM secondary electron image, (**b**–**e**) HAADF image and the corresponding EDS mapping, (**f**) HRTEM image along the 5-fold axis with the corresponding SAED pattern as the inset, (**g**,**h**) the enlarged HRTEM images along the (110) zone axes and the corresponding FFT patterns of the core (**g**) and shell (**h**) from the dotted regions in (**f**).

The original Cu_5_FeS_4_ icosahedral nanoparticles that have been stored at RT in air for more than 30 months were rechecked, in terms of morphology, chemical distribution, and crystal structures, as shown in Figure 1. The morphology and structure characterizations of fresh nanoparticles can be seen in reference [27]. As depicted in the secondary electron image of Figure 1a, the nanoparticles still possess a typical icosahedral morphology, and the corresponding EDS mapping (Figure 1b–e) results also show a core-shell structure with an Fe-rich core and Cu-rich shell. HRTEM and the corresponding SAED diffraction pattern (see Figure 1f and its inset) further confirm the twin structure of icosahedral particles. Figure 1g,h reveals the enlarged HRTEM image and corresponding fast Fourier transform (FFT) patterns of the core and shell (relate to the dotted regions in Figure 1f), respectively. As expected, a cubic structure in the shell and a relatively low-symmetry structure (i.e., superstructure of cubic phase as identified by the regular spots between main spots) in the core were confirmed, which is consistent with the results on fresh nanoparticles. Additionally, according to the measurement of *d*-spacings from HRTEM images and FFT patterns (see Figure S1 in Supplementary Materials), it was determined that the *d*-spacing of (111) planes in the core was ~3.17 Å (corresponding to *a* = ~5.49 Å referred as the *a* type structure), about 3% larger than that of ~3.07 Å (corresponding to *a*/3 = ~5.32 Å with 3*a* superstructure) in the shell. The lattice difference between the core and shell are suggested to be related to the intrinsic strain of icosahedron nanoparticles (which will be discussed in Section 3.3). Generally, according to the above characterizations and analysis, it is concluded that these non-stoichiometric Cu_5_FeS_4_ icosahedral nanoparticles have good stability at ambient temperature.

Besides the morphologies and crystalline structures, the chemical composition of particles, especially their cores and shells, was determined based on the thin slices. The average atomic ratios (on a statistical quantitative analysis over 18 particles) of Cu:Fe:S for the cores and shells (Table 1) were 45.9 (±3.1):13.7 (±2.0):37.7 (±1.3) (close to Cu_5_FeS_4_) and 63.6 (±1.0):4.4 (±0.6):31.9 (±0.7) (close to Cu_2_S), respectively. The chemical composition and structural (crystal lattice) difference between the cores and shells of these nanoparticles will be discussed in Section 3.3.

### 3.2. Thermal Stability Studies by In Situ TEM

**Figure 2:**
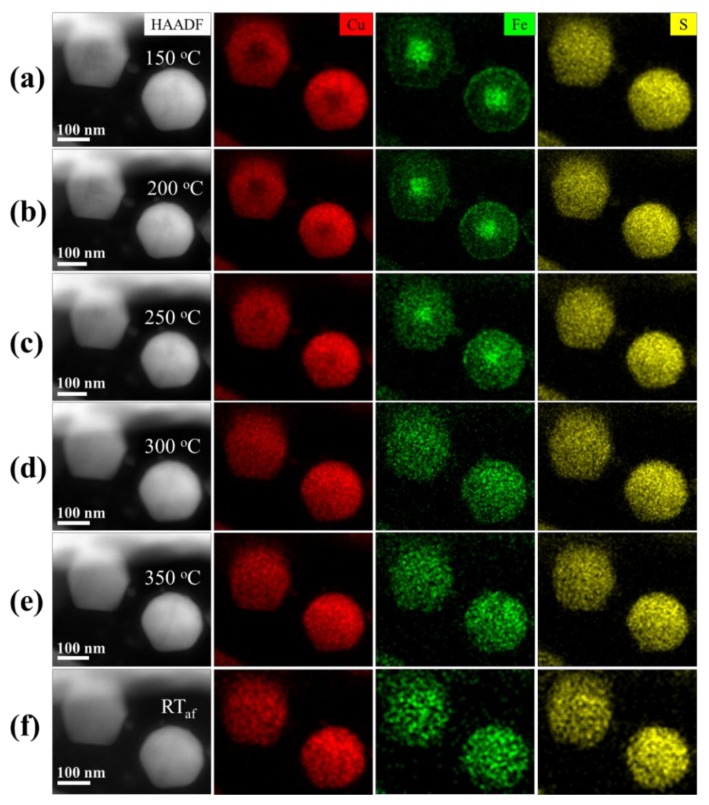
In situ chemical evolution of Cu_5_FeS_4_ nanoparticles during thermal annealing. The groups of HAADF images and the corresponding EDS mappings for Cu_5_FeS_4_ nanoparticles annealed at (**a**) 150, (**b**) 200, (**c**) 250, (**d**) 300, (**e**) 350 °C, and (**f**) returned to RT, respectively.

**Figure 3:**
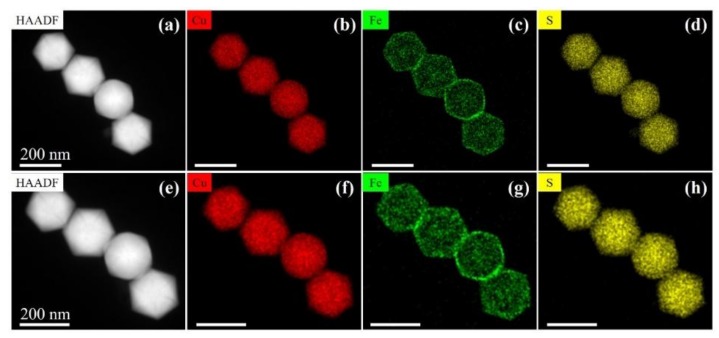
Chemical identification of Cu_5_FeS_4_ nanoparticles in air under 350 °C treatment for 1 h. The characterization of HAADF images and the corresponding EDS mappings of (**a**–**d**) newly annealed particles and (**e**–**h**) after about 50 d stored at RT, respectively.

The thermal stability of the Cu_5_FeS_4_ nanoparticles was then investigated by means of in situ heating TEM. Figure 2 displays the chemical composition evolution of Cu_5_FeS_4_ nanoparticles during in situ heating. Each group of EDS mapping was collected after keeping the particles at respective temperatures for about 10 min (see the temperature procedure in Figure S2). As revealed in HAADF images, the morphology of nanoparticles did not show a noticeable change during the whole aging process, indicating that the icosahedral morphology remained stable against thermal heating. When the annealing temperature was below 200 °C, core-shell structures with different compositions were still observed (Figure 2a,b). In contrast, because of element interdiffusion, the composition difference between the core and the shell gradually disappeared with increasing heating temperature from 250 to 350 °C (Figure 2c–e). When the temperature was returned to RT, the nanoparticles with a homogeneous composition distribution were unchanged (Figure 3f). Therefore, the chemically varied core-shell structure of the nanoparticles will easily disappear after sintering at high temperature, as observed in our previous research [27].

Comparing to the annealing in vacuum (in TEM), another thermal treatment of such nano-particles at 350 °C for 1 h in air was also performed, and generally the homogenization of core-shell structure also took place (see Figure 3a–d), which was consistent with the in situ TEM results (Figure 2e,f). Additionally, Fe-rich surfaces present (Figure 3c) after thermal annealing in air should be due to slight surface oxidation of Fe when the nanoparticles were exposed to an oxygen-rich atmosphere at high temperature (Figure S3). Moreover, after about 50 d stored in air at RT, the chemical composition of these heated nano-particles was still uniformly distributed without any signature of reformation of core-shell structures (Figure 3e–h).

**Figure 4:**
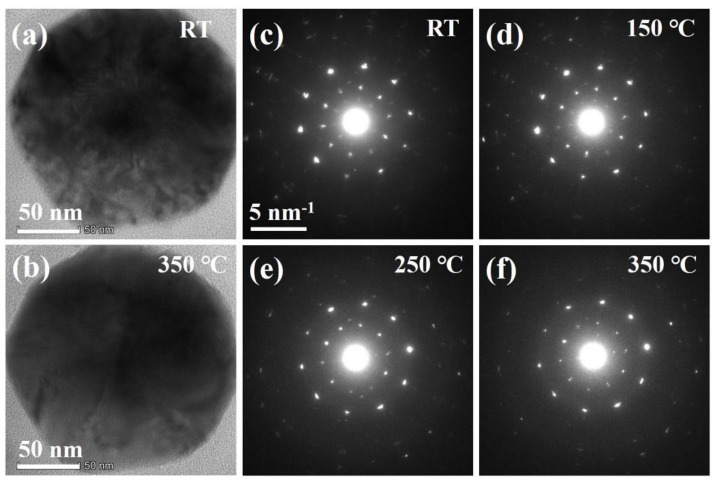
In situ TEM studies of the twinned and core-shell structures of Cu_5_FeS_4_ nanoparticles during annealing. (**a**,**b**) TEM images of nanoparticles at RT and 350 °C, respectively. (**c**–**f**) The SAED patterns collected at RT, 150, 250, and 350 °C, respectively.

The twinned and core-shell structures were also investigated (Figure 4) during thermal annealing. Figure 4a,b demonstrates the morphology of nanoparticles under room temperature and during heating at 350 °C, respectively. No significant differences in the morphologies of nanoparticles under RT and 350 °C were noticed, except that some irregular fringes in the RT nanoparticles appeared due to their relatively strong internal strain. The 5-fold SAED patterns (Figure 4c–f) confirm that the twinned structures were preserved during the whole process even after chemical homogenization (under 350 °C annealing). Additionally, some weak spots between/around the main spots almost disappeared in the SAED patterns of 250 (Figure 4e) and 350 °C (Figure 4e) samples when compared to those of RT (Figure 4c) and 150 °C (Figure 4d) nanoparticles, which should be attributed to the phase transitions. It is reported that Cu_5_FeS_4_ usually undergoes two phase transitions, that is, from orthorhombic phase (*Pbca*) to intermediate cubic phase (*Fm*$\bar{3}$*m*) at around 200 °C and then transformed into high cubic phase at about 270 °C [20]. As lattice symmetry gradually increases from orthorhombic to (high) cubic phase, the weak diffraction spots of the low-symmetry structure or superstructures will disappear.

**Figure 5:**
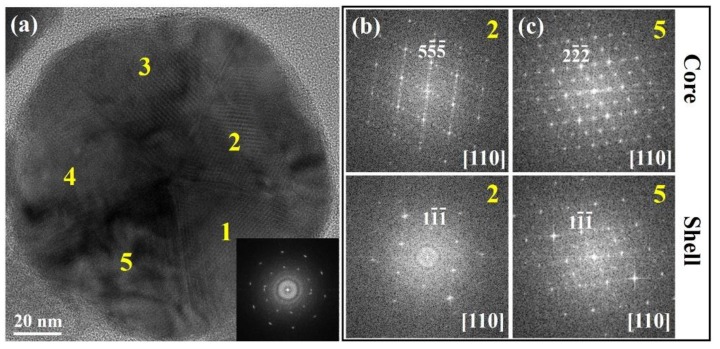
(**a**) HRTEM image and the corresponding FFT pattern (as the inset) of an annealed particle (350 °C, 1 h). (**b**,**c**) FFT patterns of the cores (upper images) and shells (lower images) from parts 2 and 5 in (a).

Finally, in order to determine the crystal structure changes of nanoparticles after chemical composition homogenization, the nanoparticles were heated at 350 °C for 1 h in vacuum and then sectioned to ultra-thin slices for HRTEM investigation. The HRTEM image and the corresponding FFT pattern (Figure 5a) were similar to those of the original particles (Figure 1f). In addition, superstructures [28,29,30,31] of 5*a* and 2*a* forms appeared in the core areas of parts 2 and 5 (Figure 5b,c), respectively, while the cubic structure was also observed in the shells. Thus, the different crystal structures of the core-shell particles (or structural core-shell of these particles) were still preserved to some extent even after chemical homogenization, indicating an inconsistency between the chemical and structural evolution of these nano-icosahedrons.

### 3.3. The Formation Mechanisim of Core-Shell Structures

**Figure 6:**
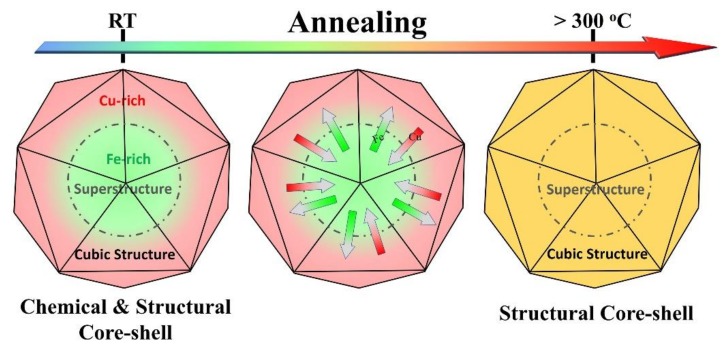
Schematic illustration of formation and evolution of the core-shell structure of Cu_5_FeS_4_ icosahedral nanoparticles.

It is reported that multiply twinned icosahedrons usually contain intrinsic strain with compression stress in the core and tension stress in the shell [32,33], where the lattice tends to gradually expand from inner to outer layers [32,34]. As mentioned above, Cu_5_FeS_4_ can crystallize into three phases: high cubic phase (*Fm*$\bar{3}$*m*, *a* = 5.49 Å), intermediate cubic phase (*Fm*$\bar{3}$*m*, *a* = 10.95 Å), and orthorhombic phase (*Pbca*, *a* = *c* = 10.95 Å, *b* = 21.862 Å) [20,29]. Moreover, the Cu_5_FeS_4_ structure can also be regarded as a modified cubic α-Cu_1.8_S (Fm$\bar{3}$m, *a* = 5.58 Å [35]) lattice wherein some cation (Cu) sites are occupied by Fe atoms and vacancies [19,28,29,30]. Therefore, it has been reported by a series of works that there are several superstructures in Cu_5_FeS_4_ and the related system, such as 2*a*, 3*a*, 4*a*, 5*a*, and 6*a* superstructures, resulting from the (disordered or ordered) arrangement of Cu, Fe, and vacancies [29,30,31]. Notably, the lattice parameter of Cu_1.8_S (*a* = 5.58 Å) was ~2% larger than that of Cu_5_FeS_4_ (*a* = 5.49 Å). As mentioned in Section 3.1 (see Figure 1g,h), the lattice parameter of the shell, *a* = 5.49 Å, was about 3% larger than that of the core, *a/3* = 5.32 Å, while the averaged Cu:Fe:S atomic ratios of cores and shells were close to Cu_5_FeS_4_ and Cu_2_S/Cu_1.8_S, respectively. By considering the errors of *d*-spacing measurements and EDS quantification, they should correspond to Cu_5_FeS_4_ (in the core) and Cu_1.8_S (in the shell), respectively. Driven by the intrinsic strain of icosahedrons, Cu_5_FeS_4_ with a smaller lattice prefers to stay in the cores, while Cu_1.8_S having a larger lattice tends to appear in the shells, which is consistent with TEM investigations. It is believed that the excellent lattice match between the core and the shell and the intrinsic strain existing in the icosahedron is crucial for the stability of the nanoparticles at ambient temperature. Therefore, the formation of core-shell structure for the original particles mainly is due to the different crystal structure and intrinsic strain of the icosahedrons and Cu_1.8_S–Cu_5_FeS_4_ system, illustrated in the left panel of Figure 6.

With temperature increase, atom motion and diffusion become stronger, leading to the chemical homogenization of the core-shell structure. After chemical homogenization (e.g., annealed at 350 °C), the nano-icosahedrons, twinned structure, and intrinsic strain of icosahedrons can be well maintained. As mentioned above, there are several structure variations (i.e., superstructures) in the Cu_5_FeS_4_ system, and the lattice parameter of the cubic structure is slightly larger than that of the superstructures, such as *a* = 5.49 Å in the high cubic phase and *a*/2 = 5.47 Å in the intermediate cubic phase. Therefore, different crystal structures tend to be preserved in the cores and shells to adapt to the intrinsic strain of icosahedral particles. That is, as seen in the TEM observations (Figure 5), although the chemical distribution became homogenous when annealed, different structures in the cores and shells were preserved to some extent because of the existence of intrinsic strain of icosahedrons, as illustrated in the right panel in Figure 6. Such remaining twin boundaries and crystal structure differences in the annealed nanoparticles (which will introduce intensive phase boundaries) are expected to enhance phonon scattering, which should be a critical reason for thermoelectric property improvement in the previous study [27].

It is of interest to note that intrinsic inhomogeneous strains are reported to exist in small particles (especially the multiply twinned particles) [36,37], which will induce chemical segregation [34,38], structural fluctuations and phase instability [39], and so on. On the other hand, the intrinsic strains of nano-particles can be utilized to improve the stability of core-shell structures and regulate crystal structures, which may be used to design core-shell and other exotic nanostructures.

## 4. Conclusions

In summary, the thermal stability and microstructure evolution of core-shell Cu_5_FeS_4_ icosahedrons were studied by in situ TEM. At ambient temperature, the particles had good stability even after exposure to air for over 30 months. In contrast, the chemical and structural thermal stabilities exhibited different behaviors under heating, as the different crystal structures can be still preserved beyond homogenized chemical composition. In detail, the core-shell structures with different chemical compositions can exist below 200 °C, while they completely disappear above 300 °C. However, the twinned structure and different crystal structures between core and shell can be maintained even after 350 °C annealing for 1 h. The relative better structural thermal stability and crystalline core-shell structure are attributed to both the intrinsic strain in icosahedral configurations and the various crystal structure variants of the Cu_5_FeS_4_ material system. These findings are not only critical for understanding the mechanism of thermoelectric performance enhancement of Cu_5_FeS_4_ icosahedral nanoparticles, but also valuable for in situ stability studies of other related core-shell nanostructures and for designing new core-shell and other exotic nanoparticles.

## Figures and Tables

**Table 1:** Chemical compositions of the cores and shells of Cu_5_FeS_4_ nanoparticles.

Core				Shell		
No	Cu (at%)	Fe (at%)	S (at%)	Cu (at%)	Fe (at%)	S (at%)
**1**	51.5	11.6	36.9	63.4	4.8	31.8
**2**	52.3	11.1	36.6	63.3	4.7	32.0
**3**	48.6	14.4	37.0	63.8	3.9	32.3
**4**	46.1	15.1	38.4	63.0	4.6	32.4
**5**	46.5	15.1	38.4	60.2	5.8	34.0
**6**	44.0	16.6	39.4	64.4	4.3	31.3
**7**	49.5	13.3	37.2	64.0	4.6	31.4
**8**	52.4	11.5	36.1	64.6	4.6	31.1
**9**	49.5	13.3	37.2	63.6	4.8	31.6
**10**	46.8	15.0	38.2	64.6	4.0	31.4
**11**	48.3	13.9	37.8	64.6	3.7	31.8
**12**	45.1	157	39.2	63.9	3.7	32.4
**13**	41.8	18.3	39.9	63.3	4.4	32.4
**14**	51.6	12.2	36.2	63.8	4.4	31.8
**15**	49.9	11.9	38.2	64.5	4.2	31.3
**16**	50.1	12.4	37.5	63.8	3.6	32.6
**17**	52.6	11.6	35.8	63.6	4.5	31.9
**18**	47.7	14.0	38.3	63.5	5.0	31.5

## Supplementary Materials

The following are available online at https://www.mdpi.com/2079-4991/10/1/4/s1. Figure S1: Measurement of lattice parameters of core and shell based on the corresponding FFT patterns; Figure S2: The heating progress for in situ TEM study. Figure S3: Chemical identification of Cu_5_FeS_4_ nanoparticles in air under 350 °C heating for 1 h.
