# Supplementary material for: Structural Core-Shell beyond Chemical Homogeneity in Non-Stoichiometric Cu5FeS4 Nano-Icosahedrons: An in Situ Heating TEM Study
Source: Nanomaterials (Basel). 2019 Dec 18;10(1):4. doi: 10.3390/nano10010004 (PMC7022726; doi:10.3390/nano10010004)
Supplement: Supplementary file 1 [file nanomaterials-10-00004-s001.pdf]

Supplementary Materials

Structural core-shell beyond chemical homogeneity in non-stoichiometric Cu_5FeS_4 nano-icosahedrons: An *in-situ* heating TEM study

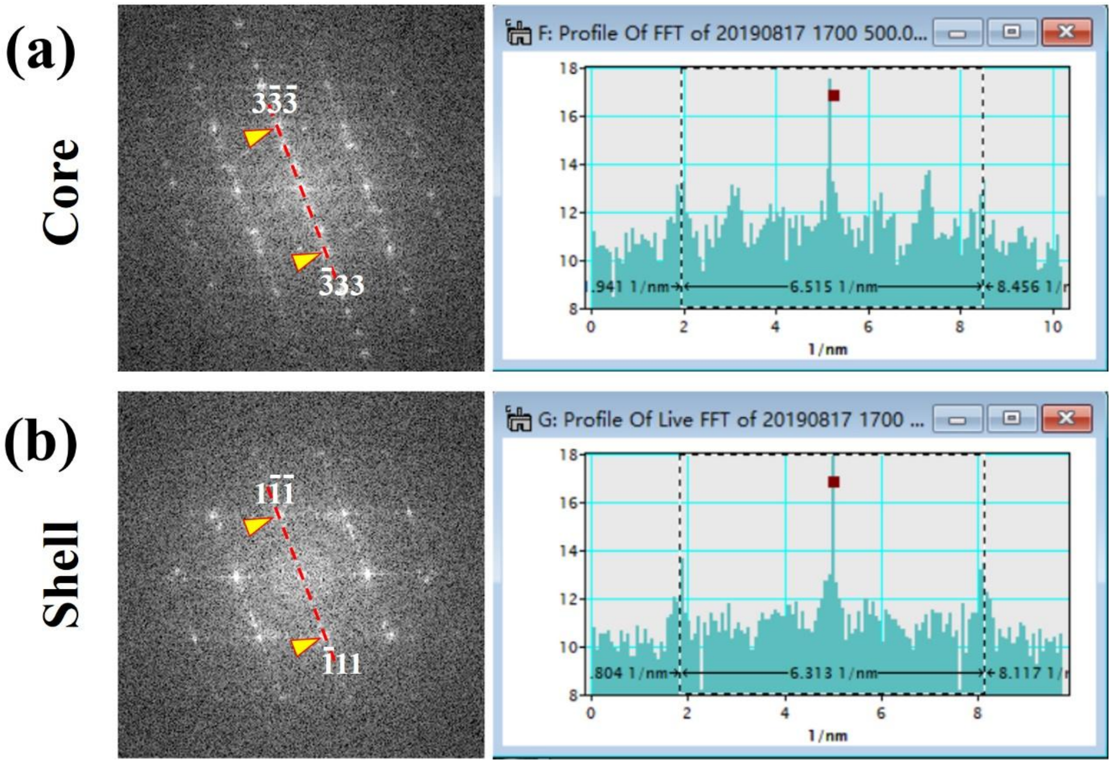

Figure S1. Measurement of lattice parameters of core (**a**) and shell (**b**) based on the corresponding FFT patterns.

Core: The *d*-spacing is $d_{(333)}$ = 2/6.515 nm^{-1} ≈ 3.07 Å; the lattice parameter for cubic structure is *a/3* = 3.07*$\sqrt{3}$ Å = 5.32 Å.

Shell: The *d*-spacing is $d_{(111)}$ = 2/6.313 nm^{-1} ≈ 3.17 Å; the lattice parameter for cubic structure is *a* = 3.17*$\sqrt{3}$ Å = 5.49 Å.

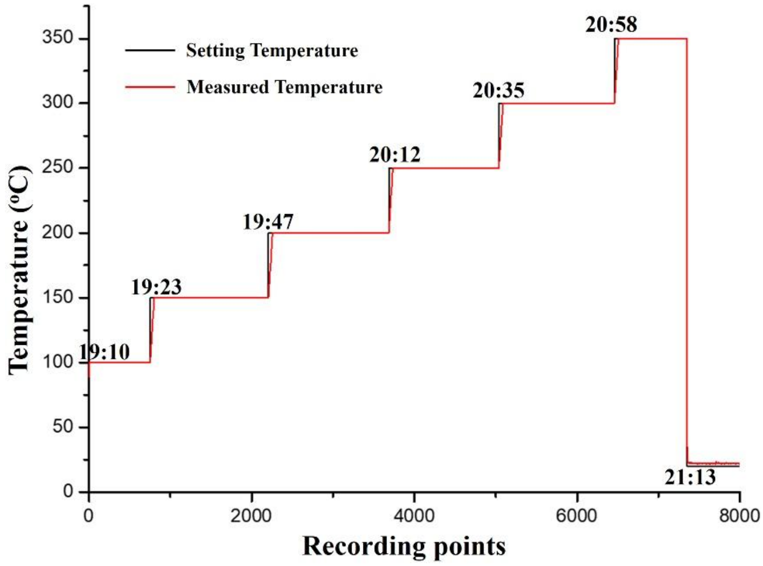

Figure S2. The heating process for *in-situ* TEM study.

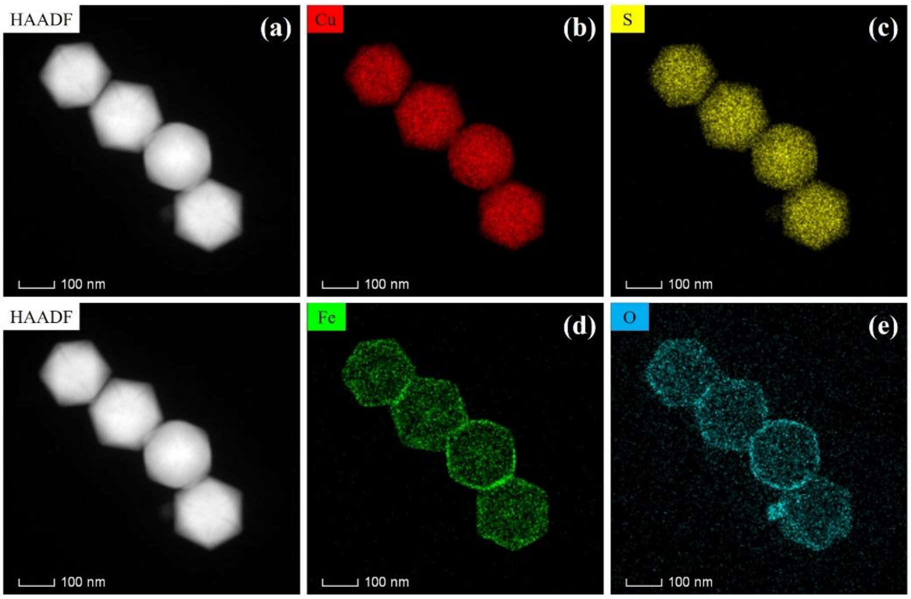

Figure S3. Chemical identification of Cu_5FeS_4 nanoparticles in air under 350 °C heating for 1 h.
